# Influenza in Pregnancy: From the Maternal Lung to the Placenta and Fetus

**DOI:** 10.3390/diagnostics16193250

**Published:** 2026-10-08

**Authors:** Radu Vlădăreanu, Andreea Borislavschi, Emanuel Moisă, Mihaela Boț

**Affiliations:** 1 Department of Obstetrics and Gynaecology, Elias Emergency University Hospital, 17 Mărăști Boulevard, 011461 Bucharest, Romania; 2 Department of Obstetrics, Gynecology and Neonatology, “Carol Davila” University of Medicine and Pharmacy, 37 Dionisie Lupu Street, 020021 Bucharest, Romania; 3 Department of Anesthesiology and Intensive Care, Elias Emergency University Hospital, 17 Mărăști Boulevard, 011461 Bucharest, Romania; 4 Department of Anesthesiology and Intensive Care, “Carol Davila” University of Medicine and Pharmacy, 37 Dionisie Lupu Street, 020021 Bucharest, Romania

**Keywords:** influenza, pregnancy, maternal immunity, placental dysfunction, fetal outcomes, neonatal outcomes, antiviral therapy, oseltamivir, influenza vaccination

## Abstract

Influenza in pregnancy is associated with a higher risk of severe respiratory disease and adverse maternal and perinatal outcomes, particularly later in gestation. This narrative review synthesizes evidence on epidemiology, pathophysiology, placental and fetal consequences, clinical presentation, diagnosis, antiviral management, and vaccination. Pregnancy involves dynamic immune adaptation together with reduced respiratory reserve, increased oxygen consumption, and cardiovascular changes that may amplify the effects of influenza pneumonia. Placental and fetal consequences appear to be mediated predominantly by maternal hypoxemia, fever, systemic inflammation, and hemodynamic disturbance; transplacental viral transmission is rare. Therefore, adverse fetal and neonatal outcomes are more closely linked to the severity and timing of maternal illness, placental dysfunction, and prematurity than to direct fetal infection. Prompt recognition is essential, and antiviral treatment should not await laboratory confirmation when influenza is suspected. Oseltamivir remains the preferred antiviral therapy and should be initiated as early as possible, including in patients presenting after 48 h with progressive or severe disease. Seasonal inactivated or recombinant influenza vaccination is the cornerstone of prevention, protecting both the mother and the infant through transplacental transfer of influenza-specific IgG. This integrated perspective supports vaccination, early diagnosis, and timely treatment to reduce maternal and perinatal risk.

## 1. Introduction

Influenza is one of the most common viral respiratory infections worldwide and remains an important cause of morbidity and mortality, particularly among populations at increased risk of complications. Although the clinical course is self-limited in most cases, pregnancy is associated with an increased risk of severe disease. Pregnant women are at greater risk of viral pneumonia, acute respiratory failure, intensive care unit admission, and maternal death than nonpregnant women of the same age, and infection may also be associated with adverse obstetric and perinatal outcomes, including pregnancy loss, preterm birth, fetal death, and neonatal morbidity [1,2,3,4,5].

This vulnerability is not determined solely by viral characteristics, but arises from the interaction between infection and the physiologic, immunologic, and cardiovascular adaptations of pregnancy. The consequences of maternal influenza may therefore extend beyond the respiratory compartment and involve the maternal–placental–fetal unit. In severe disease, pulmonary injury, hypoxemia, and the maternal systemic inflammatory response may affect placental perfusion and function, with potential consequences for fetal homeostasis. From this perspective, influenza during pregnancy should be regarded not only as a viral respiratory infection, but also as a disease with potential systemic consequences, whose obstetric and perinatal effects are closely related to the severity of maternal illness [1,2,6,7].

The relationship between influenza infection and pregnancy has attracted medical attention since the beginning of the twentieth century. Observations during the 1918 influenza pandemic first suggested a disproportionately severe course of disease among pregnant women, and the 1957 Asian influenza pandemic confirmed this vulnerability. Subsequently, the 2009 influenza A(H1N1) pandemic provided robust epidemiologic evidence linking pregnancy to an increased risk of severe disease, contributing to the designation of pregnant women as a priority group for influenza vaccination and early antiviral treatment [3,4,8,9,10].

This narrative review examines the distinctive features of influenza during pregnancy, from maternal physiologic and immunologic adaptations to the mechanisms through which respiratory compromise and systemic inflammation may affect placental function and fetal status. It further addresses the diagnostic approach to suspected influenza, including the role and limitations of molecular testing, as well as evidence-based antiviral treatment and vaccination. By integrating maternal, placental, fetal, and clinical perspectives, the review aims to clarify how maternal disease severity shapes obstetric and neonatal risk and to support timely diagnostic and therapeutic decision-making.

## 2. Materials and Methods

This narrative review was undertaken to synthesize clinically relevant evidence on influenza during pregnancy, with particular attention to maternal physiologic and immunologic adaptations, severe disease, placental and fetal consequences, diagnosis, antiviral treatment, and vaccination.

A focused literature search was conducted in PubMed/MEDLINE, Scopus, Web of Science, and Google Scholar for publications available from 1994 through 19 August 2026. Search terms were combined as appropriate and included “influenza”, “pregnancy”, “maternal influenza”, “severe influenza”, “influenza pneumonia”, “placenta”, “fetal outcomes”, “neonatal outcomes”, “antiviral treatment”, “oseltamivir”, and “influenza vaccination”.

Peer-reviewed original studies, systematic reviews and meta-analyses, and guidance issued by relevant professional societies and international public health authorities were considered. Priority was given to population-based cohorts, studies evaluating severe maternal disease and pregnancy outcomes, investigations of placental and maternal–fetal mechanisms, and evidence informing diagnostic testing, antiviral treatment, and vaccination during pregnancy. Landmark earlier publications were retained when they provided essential historical context or foundational evidence, particularly regarding the epidemiology of influenza in pregnancy and transplacental antibody transfer.

Reference lists of key publications were screened to identify additional relevant sources. Sources were selected on the basis of clinical relevance, study design, and contribution to the maternal–placental–fetal perspective of the review. Publications were generally excluded if they did not address influenza, did not provide evidence directly relevant to pregnancy or the maternal–placental–fetal framework of the review, addressed respiratory viral infections without extractable influenza-specific evidence, or did not report outcomes relevant to the clinical or mechanistic domains covered by the review. Duplicate reports were also excluded, while more complete or updated reports from the same study population were preferentially retained.

Search terms were combined as appropriate according to the database and topic being investigated and covered influenza and pregnancy, severe maternal disease, placental effects, fetal and neonatal outcomes, antiviral treatment, and vaccination. The principal search terms are summarized in Supplementary Table S1.

Given its narrative scope, this review did not follow a formal systematic-review protocol, no formal risk-of-bias assessment was undertaken, and no quantitative synthesis or meta-analysis was performed.

## 3. Incidence and Clinical Recognition of Influenza During Pregnancy

The frequency of influenza infection during pregnancy varies according to case definition and the method used to identify infection. In the multinational prospective longitudinal PRIME cohort, which included 11,277 pregnant women under active surveillance during influenza seasons, symptomatic reverse transcription polymerase chain reaction (RT-PCR)-confirmed influenza was identified in 310 participants (2.75%). Incidence was 88.7 cases per 10,000 pregnant-woman-months in the 2017 season and 69.6 per 10,000 pregnant-woman-months in 2018, corresponding to a risk of 0.7–0.9% for each month of pregnancy spent during the influenza season. In a subsequent serologic analysis of the PRIME cohort involving 1466 unvaccinated pregnant women from Peru and Thailand, molecular and/or serologic evidence of influenza virus infection was identified in 296 participants (20.2%); among these, 250 (84.4%) were detected exclusively through seroconversion. The marked difference between the frequency of molecularly confirmed symptomatic influenza and serologically documented infection suggests that a substantial proportion of influenza infections during pregnancy may be asymptomatic or paucisymptomatic and therefore remain clinically unrecognized [11,12].

These estimates should not be generalized to all populations, as influenza incidence during pregnancy is influenced by local virus circulation, access to testing, vaccination coverage, and the surveillance strategy used.

## 4. Pregnancy: A Unique Biological Environment for Influenza Infection

For many years, the increased risk of severe influenza during pregnancy was attributed almost exclusively to the concept of “physiologic immunosuppression of pregnancy.” This explanation is now considered insufficient. Current evidence indicates that pregnancy does not induce generalized suppression of the immune system, but rather a coordinated adaptation of immune responses required for the establishment and maintenance of pregnancy. This adaptation is essential because the conceptus is semi-allogeneic, expressing antigens of both maternal and paternal origin. The maternal immune system must therefore fulfill two apparently competing functions: maintaining immunologic tolerance toward the conceptus while preserving the capacity to control infection [13].

These adaptations are dynamic rather than uniform. Immune responses vary across gestation and differ between the maternal circulation and the maternal–fetal interface. Some pathways are downregulated to support tolerance, whereas others remain intact or are selectively enhanced to maintain protection against pathogens. Consequently, the immunologic environment of early pregnancy differs substantially from that of late gestation, and pregnancy should not be regarded as a state of generalized immune deficiency [13,14,15].

The increased susceptibility of pregnant women to severe influenza cannot be attributed to immunologic adaptation alone. Pregnancy-related respiratory and cardiovascular changes also contribute by reducing the maternal capacity to compensate for pulmonary injury caused by viral infection. The clinical course of influenza during pregnancy therefore reflects the interaction between gestational physiologic adaptations and the maternal response to viral infection [16].

### 4.1. Respiratory Adaptations During Pregnancy

Respiratory adaptations contribute to the increased vulnerability of pregnant women during severe respiratory infections. Functional changes occur early in pregnancy, predominantly under hormonal influence, whereas the mechanical effects of the enlarging uterus become progressively more important as gestation advances. Although these adaptations support the increased metabolic requirements of the mother and fetus, they reduce the respiratory reserve available during acute pulmonary injury [17].

One of the earliest adaptations is an increase in alveolar ventilation, driven predominantly by the effect of progesterone on the respiratory centers. Increased tidal volume and minute ventilation accommodate the gas-exchange requirements of pregnancy without causing clinically significant impairment of ventilatory function under physiologic conditions [17,18].

As pregnancy progresses, upward displacement of the diaphragm by the enlarging uterus is accompanied by a reduction in functional residual capacity, primarily through decreases in expiratory reserve volume and residual volume. This change reduces the oxygen reserve available when gas exchange becomes impaired [17,19].

Maternal oxygen consumption also increases progressively in response to maternal and fetal metabolic demands. The combination of increased oxygen requirements and reduced functional residual capacity makes hypoxemia less well tolerated during pregnancy. In influenza pneumonia, parenchymal lung injury and impaired alveolar–capillary gas exchange may therefore lead to more rapid deterioration in maternal oxygenation, with consequences for fetal oxygenation and, in severe cases, progression to respiratory failure [17,19,20].

### 4.2. Cardiovascular Adaptations During Pregnancy

Pregnancy is characterized by increased plasma volume and cardiac output together with reduced systemic vascular resistance. These adaptations support maternal and fetal metabolic demands but also impose substantial hemodynamic demands on the maternal cardiovascular system [21]. From a cardio-obstetric perspective, pregnancy has therefore been conceptualized as a physiological “cardiovascular stress test”, during which limited cardiovascular reserve or an underlying unfavorable cardiometabolic phenotype may become clinically apparent [22]. Although these adaptations are well tolerated in uncomplicated pregnancy, this altered cardiovascular state may reduce the capacity to compensate for an additional acute systemic insult. In severe influenza, hypoxemia, fever, systemic inflammation, and increased metabolic demand may impose further cardiovascular stress. Experimental evidence also suggests that influenza during pregnancy can induce systemic vascular inflammation and endothelial dysfunction [16,23]. The interaction between pregnancy-related hemodynamic adaptations and these infection-associated systemic and vascular effects may therefore contribute to hemodynamic instability and, potentially, impaired uteroplacental perfusion. However, direct evidence demonstrating this mechanistic pathway in pregnant women with influenza remains limited.

## 5. Pathophysiological Mechanisms of Severe Influenza During Pregnancy

In most cases, influenza infection is controlled by an effective antiviral response and resolves without major complications. In a small proportion of pregnant women, however, infection may progress to viral pneumonia, acute respiratory failure, acute respiratory distress syndrome (ARDS), multiorgan dysfunction, and, in the most severe cases, maternal death [1,2,16].

Severe disease results from the interaction between viral replication, the maternal immune response, and the physiologic characteristics of pregnancy [16]. Pulmonary injury, hypoxemia, and systemic inflammation may extend beyond the respiratory compartment, with effects on the vascular endothelium, placental perfusion, and the maternal–fetal unit [6,7,16,23]. In this context, available evidence suggests that obstetric complications are related primarily to the extent of maternal disease and its consequences for placental function.

Although influenza A and B viruses differ in their virologic and epidemiologic characteristics, the fundamental mechanisms of infection, immune response, and immunopathology are broadly similar. The pathophysiologic mechanisms described below are therefore applicable to both influenza virus types [24,25].

### 5.1. Respiratory Epithelial Infection and Initial Lung Injury

The lung is the principal target organ of influenza infection. The virus predominantly infects respiratory epithelial cells, where viral replication causes epithelial injury, impairs mucociliary clearance, and compromises the barrier function of the respiratory mucosa [24,25,26].

Extension of infection into the lower respiratory tract may directly injure the pulmonary parenchyma and lead to viral pneumonia. At the same time, the disruption of local host-defense mechanisms predisposes to bacterial superinfection, which may further aggravate respiratory disease. Respiratory epithelial injury also provides the initial signal for activation of the host immune response [27,28].

### 5.2. Maternal Immune Response and Systemic Progression

Recognition of viral components by respiratory epithelial cells and alveolar macrophages initiates the innate antiviral response, with production of type I and III interferons, proinflammatory cytokines, and chemokines. Interferons restrict viral replication and induce an antiviral state in both infected cells and neighboring epithelial cells, whereas inflammatory mediators promote recruitment of neutrophils, macrophages, and natural killer cells. Subsequent activation of T lymphocytes and production of virus-specific antibodies complete the antiviral response [24,29].

When viral replication persists, continued recruitment and activation of inflammatory cells may amplify pulmonary injury. In severe disease, tissue damage therefore reflects the combined effects of direct viral cytopathology and the host inflammatory response, promoting progressive impairment of gas exchange and the development of hypoxemia [24,29].

Experimental studies suggest that, as inflammation extends beyond the pulmonary compartment, the response may become systemic and be accompanied by endothelial and hemodynamic alterations. During pregnancy, these changes may affect the uteroplacental circulation and represent one of the pathways through which severe maternal disease can influence placental function and the fetal environment [30,31,32].

### 5.3. Placental Dysfunction in Maternal Influenza

The placenta forms the functional interface between the maternal and fetal circulations and plays a central role in fetal homeostasis by mediating the exchange of gases, nutrients, and metabolic products and by contributing to local immune regulation. Under physiologic conditions, it helps maintain a balance between defense against pathogens and the immunologic tolerance required for pregnancy [13,33].

In maternal influenza, transplacental viral transmission is exceptional, and evidence of viral replication within placental tissue is limited. Available data support a predominantly indirect mechanism of placental injury, mediated by maternal hypoxemia, systemic inflammation, and hemodynamic alterations rather than by direct viral cytopathic effects [1,7,16,34]. However, the evidence supporting these pathways is heterogeneous and derives from different levels of investigation, including population-based and clinical observational studies, human placental histopathology, and experimental animal models. These sources should not be interpreted as providing equivalent levels of mechanistic evidence.

Maternal hypoxemia and systemic inflammation may impair endothelial function and uteroplacental microcirculation, reducing perfusion of the intervillous space and the efficiency of maternal–fetal exchange. In parallel, proinflammatory mediators implicated in influenza-associated placental and vascular responses include interleukin (IL)-1β, IL-6, and tumor necrosis factor (TNF)-α. Experimental studies have demonstrated increased concentrations of these cytokines in placental tissue following maternal influenza A virus infection, while IL-1β and TNF-α have also been implicated in the systemic vascular inflammatory response associated with endothelial dysfunction [30,34]. Increased exposure to these and other inflammatory mediators may contribute to altered placental vascular homeostasis, barrier function, and local mechanisms of immune regulation. These changes may reduce the capacity of the placenta to adapt to hypoxic stress and compromise its function as an organ of exchange [6,30,34].

Maternal systemic inflammation may also induce a biologic response within the placenta even in the absence of detectable influenza virus in placental tissue. Experimental data have demonstrated activation of type I interferon pathways and altered expression of proinflammatory mediators, suggesting that the placenta can respond to inflammatory signals generated by maternal infection independently of transplacental viral transmission [34,35]. Human histopathologic studies have described inflammatory lesions in some cases, including chronic villitis, as well as vascular and placental perfusion abnormalities. These findings are inconsistent, however, and are not specific to influenza infection. Importantly, the available human placental evidence is based largely on limited histopathological observations and small series, which restricts causal inference and precludes definition of a specific influenza-associated placental phenotype. Moreover, the presence of inflammatory placental lesions in the absence of detectable virus in placental tissue supports a mechanism predominantly mediated by the maternal response rather than direct placental infection [34,36].

By contrast, population-based and clinical cohort studies provide evidence for associations between maternal influenza, particularly severe disease, and adverse pregnancy outcomes, but their observational design does not establish the specific placental mechanisms underlying these associations. More detailed mechanistic evidence is derived predominantly from experimental models, and its direct applicability to human pregnancy remains uncertain.

From a pathophysiologic perspective, the placenta therefore constitutes the interface through which severe maternal illness may affect the fetal environment. Available data suggest that fetal compromise is related predominantly to the extent of maternal disease and its consequences for placental perfusion and function, rather than to direct viral transmission to the fetus [1,7,16,36].

### 5.4. Fetal Consequences of Maternal Influenza

The fetal consequences of maternal influenza are influenced by disease severity and gestational age at the time of infection. Mild, self-limited illness does not appear to have the same impact on pregnancy outcomes as severe disease [2,37].

During the first trimester, maternal fever, particularly when high and persistent, is an important consideration. Epidemiologic studies have associated maternal hyperthermia with an increased risk of certain congenital anomalies, including neural tube defects. This association should not be interpreted as evidence of a direct teratogenic effect of influenza virus. Hyperthermia itself may contribute to this risk, and its impact may vary according to fever intensity and duration, timing of exposure, and use of antipyretic treatment [38,39].

During the second half of pregnancy, fetal consequences are related primarily to the severity of maternal illness. In severe disease, fetal compromise may occur and may warrant intensified maternal and fetal surveillance [1,2,7,16,40].

One of the most severe outcomes reported in association with maternal influenza is stillbirth. Observational studies and population-based analyses have identified an increased risk of fetal death in pregnancies complicated by influenza, with a stronger association during pandemic periods and in severe maternal disease. This association is considered predominantly indirect rather than a consequence of direct fetal infection and should be interpreted cautiously, because the absolute risk remains low and available studies differ in their definitions of exposure, disease severity, and the maternal and obstetric factors included in risk adjustment [40,41].

Preterm birth is another perinatal outcome reported more frequently in the setting of severe maternal influenza. It is important to distinguish spontaneous preterm birth from medically indicated preterm delivery for maternal or fetal indications. In severe disease, part of the excess risk of prematurity may reflect the need for obstetric intervention because of maternal or fetal deterioration rather than a direct effect of infection on the initiation of labor [1,2,11,37].

Evidence regarding impaired fetal growth is less consistent. Low birth weight, reported in some studies, cannot in itself be regarded as evidence of fetal growth restriction, because it may primarily reflect prematurity [11,40].

### 5.5. Neonatal Consequences of Maternal Influenza

The consequences of maternal influenza may extend into the neonatal period; however, the impact on the newborn is determined largely by the severity of maternal illness and gestational age at birth. In severe disease, the need for preterm delivery means that prematurity and its associated complications represent an important component of neonatal morbidity. Adverse neonatal outcomes reported in association with maternal influenza should therefore also be interpreted in relation to prematurity rather than automatically attributed to a direct viral effect on the newborn [2,37,40].

Neonatal influenza infection can occur. After birth, respiratory exposure to the mother, household members, or other infected individuals represents the principal route of transmission. The first months of life constitute a period of particular vulnerability because of immune immaturity and because influenza vaccination cannot be administered before the recommended age [42,43].

The proposed maternal–placental–fetal pathway through which severe influenza may contribute to adverse perinatal outcomes is summarized in Figure 1.

## 6. Clinical Manifestations and Severe Influenza During Pregnancy

The clinical presentation of influenza during pregnancy does not fundamentally differ from that in the general population. Onset is typically abrupt after a short incubation period, with fever, often high, chills, headache, myalgia, fatigue, and respiratory symptoms, particularly cough, sore throat, and coryza. Gastrointestinal manifestations, including nausea, vomiting, and diarrhea, may also occur. The absence of fever does not exclude influenza infection [1,2,3,44].

Most cases are self-limited; however, pregnancy is associated with an increased risk of severe disease and respiratory complications. This risk is facilitated by the respiratory, cardiovascular, and immunologic adaptations of pregnancy, which reduce maternal physiologic reserve and the capacity to compensate during severe respiratory infection. Vulnerability becomes more pronounced as pregnancy advances, particularly in the third trimester [1,2,16,17,18,19,20,21,45].

Pneumonia is the principal respiratory complication of severe influenza. It may present as primary viral pneumonia or as bacterial superinfection. Primary viral pneumonia is characterized by progressive worsening of respiratory symptoms, with dyspnea, hypoxemia, and pulmonary infiltrates, and in severe cases may progress to acute respiratory failure and acute respiratory distress syndrome. Bacterial superinfection should be suspected particularly when fever and respiratory symptoms recur or worsen after initial clinical improvement. The bacterial pathogens most commonly implicated include *Staphylococcus aureus*, *Streptococcus pneumoniae*, and *Haemophilus influenzae* [27,46].

This vulnerability is reflected in the spectrum of complications observed among pregnant women requiring hospitalization for influenza. In the multinational Pregnancy Influenza Vaccine Effectiveness Network (PREVENT) study, 614 hospitalizations with laboratory-confirmed influenza were analyzed. Pneumonia was reported in 60 hospitalizations (10%; 95% CI, 8–12), 27 of 592 evaluable hospitalizations required intensive care (5%; 95% CI, 3–6), and sepsis was reported in 10 cases (2%; 95% CI, 1–3). Respiratory failure occurred in fewer than 1% of hospitalizations, and no maternal deaths were reported [47]. In a US population-based study spanning nine influenza seasons, 5% of pregnant women hospitalized with laboratory-confirmed influenza required intensive care unit admission, 2% required mechanical ventilation, and in-hospital maternal mortality was 0.3% [48].

Early recognition of severity is important when evaluating a pregnant woman with influenza. Progressive dyspnea, tachypnea, hypoxemia, chest pain, marked systemic illness, inability to maintain adequate oral intake, or signs of hemodynamic instability warrant prompt assessment and, depending on severity, hospitalization. Comorbidities, particularly respiratory or cardiovascular disease, diabetes mellitus, and obesity, may further increase the risk of a complicated course [3,44].

The distinctive feature of influenza during pregnancy is therefore not a different clinical syndrome, but the potential for a more severe course in the setting of reduced maternal physiologic reserve during respiratory illness. Accordingly, symptoms suggestive of influenza in a pregnant woman should be assessed early, and signs of respiratory or systemic involvement should prompt rapid identification of patients requiring close monitoring and intensive management [3,44].

## 7. Diagnosis of Influenza During Pregnancy

The diagnosis of influenza during pregnancy integrates the epidemiologic context, clinical presentation, and detection of influenza virus in respiratory specimens. During periods of influenza circulation, acute-onset fever accompanied by cough, myalgia, headache, and systemic symptoms should raise suspicion for influenza. Clinical manifestations are not sufficiently specific to reliably distinguish influenza from other viral respiratory infections, and the absence of fever does not exclude the diagnosis [1,2,3,44].

The diagnostic approach should be tailored to the clinical setting and disease severity. In an outpatient pregnant woman with uncomplicated illness and a suggestive clinical presentation during a period of influenza circulation, laboratory confirmation is not required in every case to guide treatment. When testing is indicated, rapid molecular assays are preferred over antigen-based tests. In contrast, pregnant women requiring hospitalization for suspected influenza should undergo confirmation by RT-PCR or another molecular assay, because testing has implications for etiologic diagnosis, clinical assessment, and measures to prevent nosocomial transmission [3,49,50].

Molecular methods are preferred for etiologic confirmation. Rapid molecular assays and RT-PCR detect viral RNA, have greater sensitivity than rapid antigen tests, and can identify influenza A and B viruses. Modern rapid molecular platforms have increasingly automated workflows and short turnaround times, facilitating their use in routine clinical settings. Multiplex molecular assays can simultaneously detect influenza viruses and multiple other respiratory pathogens from a single specimen, providing broader diagnostic information and facilitating rapid differential diagnosis and targeted clinical management [49,50,51].

Despite these practical advantages, access to molecular testing may still be limited by cost, laboratory infrastructure, and local availability, particularly in resource-limited settings. Rapid molecular assays may partly overcome some of these barriers by providing sensitive nucleic acid detection at or near the point of care, with some platforms generating results within approximately 15–30 min [49,50]. However, access to these technologies remains uneven, and the applicability of diagnostic strategies derived predominantly from high-resource settings may be limited in low-resource environments [44]. Where molecular testing is unavailable or cannot provide timely results, clinical and epidemiologic assessment remains particularly important, and lack of laboratory confirmation should not delay antiviral treatment when influenza is suspected during pregnancy [44,49].

Rapid antigen tests provide results quickly but are less sensitive than molecular methods. Consequently, a negative result does not exclude influenza infection, particularly when clinical suspicion remains high or influenza activity is substantial in the community. In these circumstances, persistent clinical suspicion warrants testing with a molecular assay [49,51].

The timing and site of specimen collection influence the sensitivity of molecular testing. In nonhospitalized pregnant women, upper respiratory tract specimens should be collected as early as possible after symptom onset, preferably within the first four days, when viral shedding at this site is greatest. Nasopharyngeal specimens are preferred when compatible with the assay being used. In hospitalized patients without severe lower respiratory tract involvement, upper respiratory tract specimens should likewise be obtained as early as possible. Viral RNA may remain detectable for longer periods in severe or prolonged illness [49,50].

A particular diagnostic challenge arises in hospitalized pregnant women with severe lower respiratory tract disease. A negative molecular result from an upper respiratory tract specimen does not exclude influenza when clinical suspicion remains high. In patients with respiratory failure, particularly those receiving mechanical ventilation, testing of a lower respiratory tract specimen, such as an endotracheal aspirate or, when clinically indicated, bronchoalveolar lavage fluid, may increase the sensitivity of molecular diagnosis. Persistent viral replication in the lower respiratory tract may explain discordance between a negative upper respiratory tract specimen and the presence of severe influenza pneumonia [49,50].

Influenza virus isolation in cell culture and serologic testing are not routinely used for the acute clinical diagnosis of influenza. Viral culture requires a prolonged turnaround time and therefore has limited utility for immediate clinical decision-making; it is used primarily for virologic surveillance and characterization of circulating strains. Serologic testing likewise has limited utility in individual clinical practice because diagnosis generally requires paired sera demonstrating a significant rise in antibody titer and is therefore used predominantly for epidemiologic or research purposes [50,51].

Additional investigations are guided by clinical severity and suspicion of complications. Pulse oximetry provides an objective assessment of oxygenation, and chest imaging is indicated when pneumonia or another pulmonary complication is suspected. Pregnancy should not be a reason to delay an imaging study that is necessary to establish the diagnosis or guide management [3,52].

The key diagnostic consideration in pregnancy lies in the immediate clinical implications of suspected influenza. Laboratory confirmation should not be a prerequisite for initiating antiviral treatment. In a pregnant woman with a compatible clinical presentation and supportive epidemiologic context, respiratory specimens for etiologic confirmation can be obtained concurrently with treatment initiation, without delaying antiviral administration. The same approach is justified in severe disease when the initial test result is negative but clinical suspicion for influenza remains high [3,4,49].

## 8. Treatment of Influenza During Pregnancy

Pregnancy is an indication for antiviral treatment of suspected or confirmed influenza, regardless of trimester or vaccination status. Treatment should be initiated as early as possible, ideally within 48 h of symptom onset, when clinical benefit is greatest [3,4].

Oseltamivir, an orally administered neuraminidase inhibitor, is the preferred antiviral agent during pregnancy, including in patients with severe disease or those requiring hospitalization, because of the extensive clinical experience with its use, the available maternal and fetal safety data, and its systemic absorption, which permits therapeutic drug exposure in lower respiratory tract infection. The recommended regimen for uncomplicated influenza is 75 mg twice daily for 5 days, the same dose and duration used in the general adult population. Available observational data have not demonstrated an increased risk of adverse pregnancy outcomes associated with oseltamivir exposure, and the clinical benefits of treatment outweigh the theoretical risks of medication exposure [3,4,53,54,55].

Although treatment initiated within the first 48 h is associated with the greatest benefit, presentation beyond this interval is not a reason to withhold therapy during pregnancy. Antiviral treatment remains indicated in patients who present later, particularly those with progressive or severe disease or those requiring hospitalization. This distinction is important in obstetric practice because the 48 h interval should not be interpreted as a threshold beyond which antiviral therapy is no longer beneficial [3,4,56,57].

Inhaled zanamivir may be considered an alternative when oseltamivir cannot be used. Clinical experience with zanamivir during pregnancy is more limited, and the inhaled formulation should be avoided in patients with pre-existing airway disease because of the risk of bronchospasm. Intravenous peramivir may be considered when oral or enteral administration or absorption of oseltamivir is not feasible. Data on peramivir use during pregnancy remain limited, and clinical experience is substantially less extensive than with oseltamivir. Baloxavir offers the convenience of single-dose administration, with a potential advantage for treatment adherence. However, it is not recommended during pregnancy because data on maternal and fetal efficacy and safety are insufficient. The convenience of a simplified regimen therefore does not justify the use of baloxavir in pregnant women in place of oseltamivir, for which substantially greater clinical experience is available [3,4,58].

Antiviral treatment should be accompanied by symptomatic and supportive measures appropriate to disease severity. Fever control is particularly important during pregnancy because of the potential effects of maternal hyperthermia. Adequate hydration and respiratory monitoring are important, and patients with hypoxemia, pneumonia, clinical deterioration, or other features of severe disease require hospitalization and multidisciplinary assessment involving obstetric, infectious diseases, and, in severe cases, critical care teams. Respiratory support should be tailored to the degree of pulmonary impairment and maternal clinical status [3,38,39,44].

In complicated disease, management should be individualized according to maternal and obstetric status. Suspected bacterial superinfection warrants appropriate evaluation and antibiotic therapy, without replacing antiviral treatment when influenza is suspected or confirmed. Severe disease requires a multidisciplinary approach involving obstetric and infectious diseases teams and, when necessary, critical care specialists [4,27,44,46] (Table 1).

## 9. Prevention of Influenza During Pregnancy: Influenza Vaccination

Influenza vaccination is the principal strategy for preventing influenza and its complications during pregnancy. The timing of vaccination should be considered in relation to two distinct objectives: achieving maternal protection during periods of influenza virus circulation and allowing sufficient time for development of the maternal immune response and transplacental antibody transfer. Seasonal vaccination is recommended for pregnant women regardless of gestational age, using inactivated or recombinant influenza vaccines. Live attenuated influenza vaccines are not recommended during pregnancy. Vaccination is recommended during every influenza season, including for women vaccinated in a previous season or during a prior pregnancy, because of antigenic variability among circulating viruses and the progressive waning of post-vaccination immunity [3,59].

Available evidence supports a favorable safety profile for influenza vaccination during pregnancy. A systematic review including 63 studies found no association between seasonal influenza vaccination and an increased risk of spontaneous abortion, preterm birth, delivery of a small-for-gestational-age infant, or fetal death. With regard to teratogenicity, a recent meta-analysis including more than 1.2 million pregnancies found no increase in the overall risk of congenital anomalies or major congenital anomalies following administration of inactivated influenza vaccine during the first trimester [60,61].

The benefits of maternal vaccination extend beyond prevention of infection. Immunization reduces the risk of laboratory-confirmed influenza and influenza-associated hospitalization during pregnancy, an important consideration given the increased susceptibility to severe disease in this population. Vaccine effectiveness varies between seasons according to the antigenic match between vaccine strains and circulating viruses; nevertheless, the clinical benefit of maternal vaccination is supported by randomized and observational studies conducted across different populations and influenza seasons [10,62].

An additional benefit of influenza vaccination during pregnancy is passive protection of the newborn and young infant. Vaccine-induced influenza-specific IgG antibodies are actively transferred from the maternal to the fetal circulation, a process mediated predominantly by the neonatal Fc receptor (FcRn) expressed by the syncytiotrophoblast. The efficiency of transplacental transfer increases progressively with gestational age: at 17–22 weeks, fetal IgG concentrations are approximately 5–10% of maternal levels, reaching approximately 50% between 28 and 32 weeks and rising rapidly thereafter, such that fetal concentrations at term may equal or exceed maternal concentrations. Neonatal protection therefore depends on maternal antibody titers, gestational age, and the interval between vaccination and delivery, with sufficient time required both for development of the maternal immune response and for placental antibody transfer. Preterm birth is particularly relevant because a shorter gestation limits the time available for transplacental transfer and is associated with lower maternally derived IgG concentrations in the newborn, at a stage of life characterized by heightened vulnerability to respiratory infections. After birth, maternally derived antibody concentrations in the newborn and subsequently the infant decline progressively; passive protection is therefore greatest during the first months of life and wanes thereafter. The clinical benefit of maternal immunization may persist through approximately 6 months of age, before the infant becomes eligible for influenza vaccination [63,64,65].

A potential concern regarding maternally derived influenza-specific antibodies is whether they may attenuate the infant’s immune response to subsequent active influenza vaccination, a phenomenon generally referred to as immunological blunting. However, influenza-specific human data remain limited, in part because routine influenza vaccination begins at 6 months of age, by which time maternally derived antibodies have substantially waned. Although pre-existing antibodies may reduce serological responses when influenza vaccines are administered earlier in infancy, the clinical relevance of this effect at the recommended age of infant vaccination remains uncertain [66].

The clinical relevance of this passive protection is supported by reductions in laboratory-confirmed influenza and influenza-associated hospitalization among infants born to mothers vaccinated during pregnancy. A recent observational cohort study including 245,498 infants within the Kaiser Permanente Northern California integrated healthcare system in the United States between 2011 and 2022 reported an adjusted 44.4% reduction in influenza during the first 6 months of life among infants whose mothers were vaccinated during pregnancy [67].

Additional preventive strategies are particularly relevant for young infants who are not yet eligible for influenza vaccination. Vaccination of household members and caregivers may provide an additional layer of protection by reducing the infant’s exposure to influenza [43]. Women who were not vaccinated during pregnancy should be encouraged to receive seasonal influenza vaccination postpartum, including while breastfeeding; influenza vaccination is safe during lactation and may also provide some passive protection through antibodies in breast milk [3].

Vaccination timing should be adapted to the local seasonality of influenza virus circulation, with the aim of maintaining maternal protection throughout the period of influenza activity while, when possible, allowing an adequate interval for development of the maternal immune response and transplacental antibody transfer. In temperate regions of the Northern Hemisphere, vaccination is preferred in September and October for women who are in the first or second trimester, whereas vaccination in July–August may be considered for women in the third trimester, including to enhance protection of the infant during the first months after birth. In other geographic regions, vaccination timing should be aligned with the local influenza season. Vaccination remains indicated later in the influenza season for women who have not yet been vaccinated, as long as influenza viruses continue to circulate [3,59,68].

## 10. Discussion

The evidence reviewed here supports several clinically relevant conclusions. First, the obstetric and perinatal consequences of influenza appear to be driven predominantly by the severity of maternal illness and its indirect effects on placental and fetal homeostasis rather than by direct fetal infection. Maternal hypoxemia, fever, systemic inflammation, and medically indicated preterm delivery are therefore important considerations when interpreting reported adverse pregnancy outcomes [1,2,16,37,40,41]. Second, suspected influenza during pregnancy warrants prompt clinical assessment and early antiviral treatment; laboratory confirmation may support diagnosis but should not delay oseltamivir when treatment is clinically indicated [3,4,49]. Third, seasonal influenza vaccination remains the principal preventive intervention, providing maternal protection while also extending protection to the young infant through transplacental transfer of influenza-specific antibodies [3,59,62,63,64,65,67].

These conclusions should be interpreted in light of an important limitation of the present review. As a narrative rather than systematic review, study selection was not based on a formal systematic-review protocol, and no formal risk-of-bias assessment or quantitative evidence synthesis was performed. In addition, the underlying literature is heterogeneous in study design, influenza ascertainment, disease severity, gestational timing, and outcome definitions, which limits direct comparison across studies and the strength of mechanistic inference.

## 11. Knowledge Gaps and Future Directions

Despite substantial advances in recent years, uncertainty remains regarding the specific contribution of the placental response to the fetal and perinatal consequences of maternal influenza. Available data do not yet allow the effects of viral infection to be fully disentangled from those of fever, hypoxemia, systemic inflammation, or maternal disease severity, and the placental abnormalities described to date are not specific to influenza infection [6,7,16,23,34,36].

Future research would benefit from prospective longitudinal pregnancy cohorts with standardized definitions of influenza exposure, maternal disease severity, and obstetric and neonatal outcomes. Serial maternal sampling during acute infection and recovery, including inflammatory, endothelial, and placental biomarkers, combined with standardized placental histopathology and, where feasible, molecular and transcriptomic analyses at delivery, could help define the temporal relationship between maternal infection, systemic responses, placental dysfunction, and fetal outcomes. Such studies should stratify analyses by gestational age at infection, influenza subtype, vaccination status, antiviral treatment, and maternal comorbidities and should distinguish spontaneous from medically indicated preterm birth.

The potential long-term cardiovascular implications of severe influenza during pregnancy also remain poorly defined. Pregnancy is increasingly recognized as a cardiovascular stress test, and established adverse pregnancy outcomes, including hypertensive disorders of pregnancy and gestational diabetes, are associated with increased subsequent maternal cardiovascular risk [22]. Whether the systemic inflammatory and endothelial disturbances associated with severe gestational influenza similarly identify women at increased long-term cardiovascular risk remains unknown. Longitudinal studies assessing cardiovascular and cardiometabolic outcomes after severe influenza during pregnancy are therefore needed.

Structured postpartum cardiovascular surveillance has been proposed for women experiencing established adverse pregnancy outcomes, extending cardiovascular risk assessment beyond the immediate postpartum period [22]. Whether women recovering from severe influenza during pregnancy would similarly benefit from structured cardiovascular follow-up has not been established. Given the systemic inflammatory and endothelial disturbances associated with severe infection, this possibility warrants further investigation, particularly in women with coexisting cardiometabolic risk factors or recognized adverse pregnancy outcomes.

## 12. Conclusions

Influenza during pregnancy should be viewed as more than a respiratory illness. Pregnancy-related immunologic adaptations and reduced respiratory and cardiovascular reserve may predispose to severe disease, while maternal hypoxemia and systemic inflammation may have consequences for the placenta and fetus.

Fetal effects are predominantly indirect, arising from the severity of maternal illness and impaired placental function. Early recognition and prompt initiation of antiviral therapy are essential, with oseltamivir remaining the preferred antiviral agent during pregnancy.

Influenza vaccination is the principal preventive measure, providing benefits for both the mother and the newborn and young infant through transplacental transfer of influenza-specific antibodies. Prevention and early treatment therefore contribute to reducing maternal risk and improving perinatal outcomes.

## Figures and Tables

**Figure 1:**
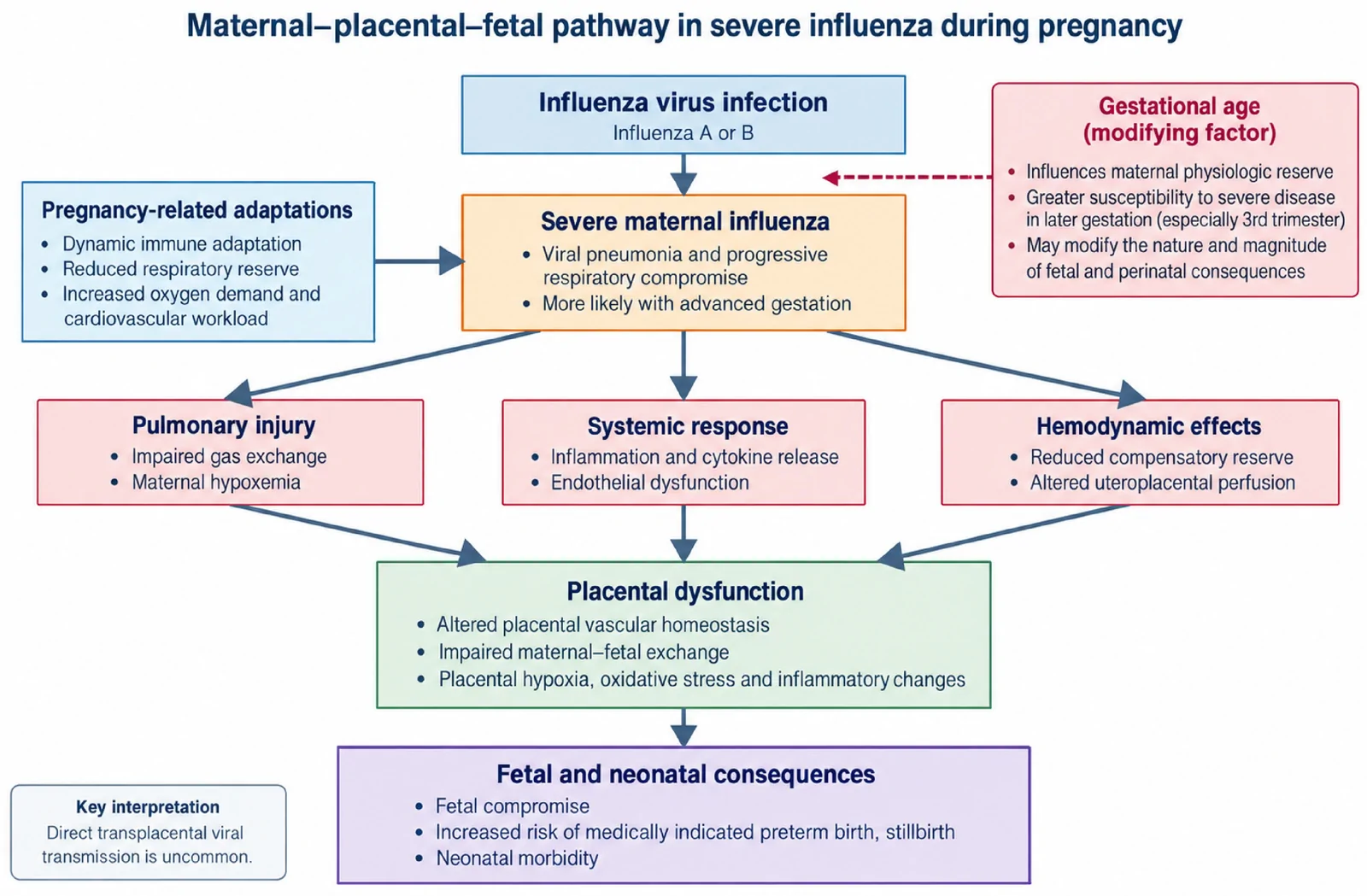
Maternal–placental–fetal pathway in severe influenza during pregnancy. Pregnancy-related immunologic, respiratory, and cardiovascular adaptations may contribute to severe maternal influenza. Pulmonary injury, hypoxemia, systemic inflammation, endothelial dysfunction, and hemodynamic alterations may impair uteroplacental perfusion and placental function, thereby contributing to fetal and neonatal compromise. Gestational age acts as an important modifying factor, influencing maternal physiologic reserve, susceptibility to severe disease, and the nature and magnitude of fetal and perinatal consequences. Direct transplacental viral transmission is uncommon. Mechanistic pathways involving endothelial dysfunction, hemodynamic alterations, and impaired uteroplacental perfusion are supported predominantly by experimental and animal data, while direct human evidence remains limited. Created by the authors based on the evidence reviewed in [6,7,16,23,30,31,32,33,34,35,36].

**Table 1:** Practical diagnostic and initial management approach for suspected influenza during pregnancy.

Clinical Setting	Diagnostic Approach	Immediate Management	Key Considerations
Uncomplicated outpatient illness [3,4,45]	Clinical diagnosis; molecular testing when clinically indicated	Start oseltamivir without awaiting confirmation	Treatment in any trimester
Hospitalized patient [3,49,50,51,52]	RT-PCR/molecular test; assess oxygenation; consider imaging	Oseltamivir; appropriate infection-control precautions; monitor for complications	Test lower respiratory tract sample if severe disease and upper test negative
Severe/critical illness [3,4,27,44,46]	Multidisciplinary assessment; evaluate pneumonia/superinfection	Antiviral therapy + respiratory/critical-care support	Maternal stabilization supports fetal oxygenation and uteroplacental perfusion

Abbreviations: RT-PCR, reverse transcription polymerase chain reaction.

## Data Availability

No new data were created or analyzed in this study. Data sharing is not applicable to this article.

## Supplementary Materials

The following supporting information can be downloaded at: https://www.mdpi.com/article/10.3390/diagnostics16193250/s1, Table S1: Search domains and principal search terms used in the literature search.
