# Supplementary material for: Influenza in Pregnancy: From the Maternal Lung to the Placenta and Fetus
Source: Diagnostics (Basel). 2026 Oct 8;16(19):3250. doi: 10.3390/diagnostics16193250 (PMC13664546; doi:10.3390/diagnostics16193250)
Supplement: Supplementary file 1 [file diagnostics-16-03250-s001.zip › diagnostics-4584918-supplementary.pdf]

Supplementary Table S1 Search domains and principal search terms used in the literature search.

Search domain	Principal search terms
Influenza and pregnancy	“influenza”; “pregnancy”; “maternal influenza”
Severe maternal disease	“severe influenza”; “influenza pneumonia”
Placental effects	“placenta”
Fetal and neonatal outcomes	“fetal outcomes”; “neonatal outcomes”
Antiviral treatment	“antiviral treatment”; “oseltamivir”
Vaccination	“influenza vaccination”
